# Her Heart's Mistake

**Published:** 1889-09-21

**Authors:** E. D. Cumming

**Affiliations:** Author of "An Ordeal by Silence


### Her Heart's Mistake.

By E. D. Cumming, Author of "An Ordeal by Silence.

(Continued from page 382.)

### Chapter XXIII.—A Discovery.

Kate's happiness in the possession of her child was short-lived. May came, and with it the heat of the Indian summer; but Bairdsley still hesitated to send his wife and son to the hills, though the delicacy of the latter had been a continual source of anxiety. He waited a few weeks too long, and one sultry, windless evening he returned home from the club to find the baby dead in its mother's arms.

Kate never fully understood how completely her husband was estranged from her until the child was taken from them. The gulf is wide that a common grief cannot bridge; but Bairdsley's sorrow, deep though it was, drew him no nearer to his wife, who was prostrated by the blow. His utter selfishness had never appeared so glaring as it did now. He shrouded himself in his own grief, regardless of her forlorn wretchedness; and Kate, who had faintly hoped that their bereavement might rekindle his old affection, was soon compelled to acknowledge to herself that it was irrevocably gone. It was the one opportunity for both; the child's death had left a void which her husband might have filled; but either he did not know it, or did not care to take advantage of it, and left Time unaided in bringing comfort to her.

Bairdsley did not allow his sorrow to interfere with his amusements, and a month after the child's death he was once more absorbed in his usual pastimes, neglecting his wife more entirely than before. She angered him by nursing her trouble for so much longer than he did, regarding its greater intensity as a reproach, and even his boon companions began to shake their heads at his remissness.

"Common decency ought to make him pay her a little more attention," said Captain Phelps, whose admiration for Bairdsley's character had not been enhanced by long acquaintance.

"No one expects him to hang round his wife incessantly; a fellow must have *some* relaxation, but; hang it all, he's never out of the club, morning, noon, or night," said Major Wilton.

"Is it true that he has been plunging lately?" asked another man from an armchair in the corner of the anteroom.

"I don't know what you call plunging, Halley; but I do know that old Sanderby took home Bairdsley's I.O.U. for nine hundred and fifty rupees at three o'clock in the morning, a little while ago—last week it was."

"Phew!" whistled the other; "that's hard going even for a man of means. Ecarté, I suppose?"

"Yes; but friend Bairdsley seems to have reached that stage of the gambling mania when he'd rather toss his bearer for two-anna bits than do nothing."

"And lick his bearer when he presumed to win," added Phelps. "I suppose it's a form of sorrow, but it's a deuced expensive shape for it to take."

"Poor Mrs. Bairdsley has taken the loss terribly to heart," said Major Wilton, after a pause.

"Is it true that she is going home?"

"Armstrong told Bairdsley that she would be the better for complete change, but I don't know whether she's going or not."

"He will send her, if that's the case."

"Don't know," said Major Wilton, with a shrug of the shoulders; "he's an incomprehensible individual."

"She has changed fearfully since her marriage," said Phelps. "At Meerut she was as lively and pleasant a girl as you could wish to meet. Now she hasn't a word to say for herself or anybody else, and has fallen off most painfully in her looks."

A profound sigh emanated from the recesses of the *Sporting Times* beneath which Mr. Wentworth's nether man was visible, but it passed unnoticed.

"She will be a great loss to the hospital if she goes home," said Captain Halley. "Every man who comes out does so with the fixed impression that Mrs. Bairdsley is the guardian angel of that dreary establishment."

"Does she go there now?" enquired Major Wilton.

"Yes; she resumed her visits a week ago on hearing from Armstrong how the men missed her, and goes nearly every afternoon.

"That's a woman to respect," said Phelps under his breath, "Not many would have laid aside such a sorrow as hers, to go and read herself hoarse to the Tommies in that dismal hole up in the fort."

He had spoken in a low tone to a man standing at his side, but all in the room heard the speech, and a murmur of approval followed it. Bairdsley's negligence looked more heartless than before in the eyes of his friends afterwards.

Kate had kept up her habit of visiting in the wards when she came to Delhi, though, of course, irregularly after she became a mother, and after her child's death she sought refuge from her grief by resuming the practice as soon as she was allowed to do so.

"You mustn't stay there long, Mrs. Bairdsley," said Dr. Armstrong; "you're not strong enough for it. I'm going to tell your husband that you ought to go home."

Kate disclaimed the necessity for leaving India, though her heart bounded at the doctor's words. The yearning to go had become more powerful than ever lately, for her father had written plaintively and sadly of his loneliness in the old country and his wish to see her again.

"You want to go and see your father very much, I've no doubt," said Bairdsley, sarcastically, when she told him Dr. Armstrong's opinion. "There's no one else you want to see in England, is there?"

"No," replied Kate, "I have very few friends there. Do let me go, George. Papa wants me, and—— "you do not," she had almost added, but checked herself in time.

"And who else?" asked Bairdsley, cynically.

Kate made no reply; she understood what he meant.

"You didn't think it likely you might see Mr. Warren," he shouted coarsely across the table. "Of course you've never had a line from Mr. Warren since you married me. You never hear from *him?*"

"No," said his wife, with quiet dignity. "I have never heard from him."

"You never found a letter from him addressed to you at the hospital by accident, I suppose?"

Kate rose from her chair and confronted him with flashing eyes, while the colour mounted to her cheeks with shame at the imputation.

"You aren't prepared to deny it, I see. Ha-ha! Thought I'd never find it out."

He laughed again as he spoke, but she did not answer him. She thought he had been drinking, and meant to leave him to recover himself alone.

"No," she said, disdainfully. "I will not deny such a charge."

His look of sneering disbelief was thrown away, for she turned her back upon him as she answered, and went into her own room, where she sat down to consider what she ought to do. It was impossible for her to continue this existence with Bairdsley. She must escape from him somehow, and if he would not allow her to go home the only alternative was a hill station.

He looked into her room on his way from the dining-room to the verandah, and told her that she might dismiss her idea of going to England at once.

"I saw Armstrong this morning," he said, "and as he says you want change, you may go to Simla, and stay there as long as you please."

"Won't you let me go home, George," she asked earnestly; "pray let me go and live with papa."

"No, I won't. That's final, and you needn't bother me any more about it."

He left the room, and a few minutes later she heard him walking down the path towards the compound gate; she waited until the sound of footsteps died away, and then calling her ayah to help, began preparations for immediate departure. He had accused her of maintaining clandestine correspondence with Warren, and the base cruelty of the charge stung her more deeply than any taunt he had ever levelled at her. For the first time since her wedding day she to-night asked herself whether she had done right in keeping her word to him. Surely she was reaping a punishment that only the blackest sin could deserve.

She rose early, as usual, next morning, and busied herself packing. Bairdsley had not come near her since the previous evening, but she heard him come in from parade, take off his sword, and throw himself into a long chair in the verandah. He had not been in very long when she heard the voice of a European begging some favour from him; he answered his visitor gruffly at first, but presently gave an impatient exclamation and called to her to come to him.

"Are there are old clothes of mine I could give this man?" he asked, nodding towards a private in the uniform of the 110th, who stood to "attention" before him. "Can you find an old suit to give him?"

"Where shall I look?" asked Kate.

"There are some clothes I wore up at Simla which I sha'n't require again; you might give him those. I think they are in the bottom drawer of the chest in my dressing-room."

He told the soldier to wait, and Kate went to search for the clothes. Bairdsley's habits were not methodical, and it took her some time to find the articles. She knelt down before the open drawer and heaped its contents on the floor until she discovered a suit of tweed which had been a good deal worn, and which she guessed were the clothes he referred to. She laid them aside and replaced the other things; then she stood up to examine those she had kept out.

"I must look through the pockets before I give them away," she said to herself. "He is so careless that he may have left anything in them."

She felt through the coat pockets first, but felt nothing except a letter unprotected by an envelope, which she placed on the dressing-table, and continued her search. As she thrust her hand mechanically into one pocket after another without finding anything, her eyes rested, unconsciously at first, upon the paper she had laid on the table. The handwriting was remarkable—singularly upright, bold, and legible; and as she looked at it, she suddenly became aware that her interest had merged from study of the caligraphy to the import of the words.

"Your intended is heiress to—"

What was this? The garment she held dropped from her hands, and she stood transfixed with astonishment. For fully five minutes she hesitated; she recognised the handwriting of the letter now, it was one not easily forgotten; the epistle George had received one day last November, which had given him so much annoyance, was from the same person. "Your intended is heiress to;" Kate's was not a curious disposition, but the words told her that this letter contained matter concerning herself; it was long before she could make up her mind to read it, but eventually she cast scruple aside, and unfolded the paper. It was dated eighteen months back, and had evidently been received by her husband when he was in Simla, just before their marriage.

"My dear Bairdsley (it ran),

"I congratulate you most heartily upon the success of your bold stroke. It deserved success. You were in luck to find the quarry still at Meerut, as I understand that Army doctors are bucketed about the country just as much as soldiers by a paternal Government. However, to business. I am now able to tell you that Miss Carsworth has given her will into our custody, and that further alteration is improbable. Its provisions are precisely what I told you. Your intended is heiress to every shilling the old lady possesses, barring a few hundreds to charities and servants, which you will not grudge out of a hundred and twenty thousand. The money is safely invested in various concerns, which I needn't detail. There is only one trifling risk you have to fear; so slight that it's hardly worth mentioning, but as Miss Carsworth is a garrulous old party, and talks to me pretty freely, I may as well mention it. She is, as you know, a second cousin of Dr. Harrison's; he and his daughter are the only relatives she has in the world, and she sometimes talks of altering the will and giving him a life interest in the property. I have dissuaded her so far; fortunately for you her hand is paralysed, and she can't write. Correspondence between her and Dr. Harrison might have militated very seriously against your interest. I don't think the old lady can last many years; she goes to Cannes every winter, but bronchitis will carry her off before long, when your wife will come into her own. That fellow Samuels was here again to-day, and had some unpleasant things to say about you. I calmed him with a sight of your letter to me and Miss Carsworth's will, so you need fear no trouble from him now. He had intended to go down to Surrey to see if your governor would be inclined to buy some little pieces of stamped paper, upon each of which your signature has a place. So sorry to hear of your illness. With kind regards and renewed congratulations,

"Yours, very sincerely,

"ROBERT ANDERSON."

"You're a precious long time finding that suit," said Bairdsley, coming into the room just as she reached the end. "What are you doing with that?" he asked sharply, seeing the letter in her hand.

She gave it to him without a word, and stood looking him full in the face.

"Have you ——?" But he stopped; he saw she had read it, and sat down on the edge of the table, leaving his question unfinished. He folded his arms and tried to look defiant and careless; but he could not meet her eyes, and sat gazing at the floor in silence.

"I will leave your house to-morrow and never enter it again," she said, turning to leave the room. "Don't touch me," she cried, as he rose and extended his hand to detain her. She shrank from him as from the touch of a leper, and fled into her own apartment, unheeding his calls. He did not attempt to follow her; she knew his secret now, and could cast off his authority if she pleased. He picked up his lawyer's letter and glanced at it; how bitterly ironical Anderson's congratulations looked! Miss Carsworth's fortune was further from him than it had been before he married Kate; he knew she was in earnest when she said she would never enter his house again. He would never see a penny of that £120,000 now. What madness had allowed him to leave that letter in his pocket, and send her to look out the very suit in which it lay? He ground his teeth with chagrin as he tore it into fragments. What was he to do next? Hardened as he was, he could not face her after this. She must be allowed to do just what she chose. It could make no difference now whether she went to England or not. Until that letter opened her eyes she did not even know of Miss Carsworth's existence, for he had sounded her most carefully before he even proposed to her. He had run himself heavily into debt before he came out to be able to settle a few thousand pounds upon her by way of a blind, and by this miserable little oversight of leaving a compromising letter undestroyed his whole elaborate scheme had been revealed. He had loved her when he first knew her, honestly loved her, and had been ashamed of the motive which had brought him out to India, then. But he thought that this lawyer Warren had come out on the same mission, and when Kate's petition to be set free reached him, he saw only a competitor in the race for a pecuniary prize.

The stake was great; his vanity and cupidity were both roused, and he had told himself that his love, such as it was, justified him in marrying her against her will. He had intended to set himself earnestly and truly to regain the affection he believed she once entertained for him after their marriage; but the change in her whole temperament soon wore out his short patience, and, confident that when she inherited her cousin's fortune, it must benefit him whether she wished it or not, he let himself go, and gave free vent to his true nature. And this accident had parted them too completely to leave room for hope of reconciliation.

### CHAPTER XXIV.—UPON THE HILL.

WHEN Kate left her husband's presence she did so intending to alter her destination from Simla to England, and leave for Bombay by the afternoon mail. But she reflected that if she decided to go home it would be impossible to get away at once; there would be great difficulty in obtaining at a moment's notice a passage in any respectable vessel at this season of the year, and a longer or shorter delay would be inevitable. Whereas if she went to the hills she could go that evening; and once there could correspond unrestrainedly with her father, and obtain his advice as to the course she should pursue.

Having resolved upon this she waited until she heard Bairdsley going out, and came into the verandah to intercept him.

"I shall leave for Umballa this evening," she said.

"Very well," he answered, indifferently; "I'll be back in time to see you off."

He did not wish his domestic embroilments to become public property, and accordingly let it be known at the club that he was going away so much earlier than usual in order to take his wife to the station. There he purchased tickets for her and her servants, saw the baggage weighed and labelled, and performed other small services with assiduous attention.

He stepped into the carriage just before the train started as if with the intention of saying something, but she gave him no encouragement, and when the bell rang he turned to go without having spoken.

"Good-bye," he said quietly.

Kate returned his farewell without raising her eyes to look at him; he had not offered to shake hands, much less embrace her; she was glad to have been spared that formality; the atmosphere seemed purer when he had gone.

She reached Umballa before daylight the next morning, after a long night's journey, which heat and dust, added to her own troubles, rendered sleepless and tedious. She took up her quarters in The Empress Hotel, and retired at once to rest before continuing her journey that afternoon. The novelty of travelling by dâk gharry wore off after the first few stages, and the sameness of the level road offered nothing to distract her thoughts. One change of horses after another at every fourth mile was accomplished with half a minute's delay at each, until the grand magnificence of the Himalayan snows became visible through the haze. The dreamlike beauty of the glistening peaks and ridges gave her new sensations, and for the first time since she left Delhi she forgot her sorrows, and lost herself in breathless reverential awe. Those faraway snow-clad heights belonged to another world in their unsullied purity; they were steps to heaven which human foot might not defile.

The wide stony bed of the Gugger was crossed, a team of huge white bullocks having replaced the shaggy country-breds. The river was low now, but the waters lapped and sucked at the flooring of the gharry, and foamed amongst the wheels as though striving to carry it bodily away. Horses were substituted for the slow-paced cattle when the stage bungalow on the far side was reached, and the Punjaubee driver hallo'd them up the slope; the first slope of the hills. The snows were out of sight now, and Kate wedged herself into a corner while the clumsy conveyance jolted and plunged over the rough road as the sturdy pair which drew it faced the steep incline at a gallop. Kalka at last; gladsome sight to the dusty traveller, who must perforce rest there until daylight allows the dangerous precipices of the hill road to be encountered. The sun had set when the gharry turned into the hotel gates, for Kate had not left Umballa until the afternoon. She was worn out with the twenty mile drive, and, even had regulations allowed it, she could not have faced the hill-road that night. Here, two thousand feet above the sea level, the evening air was cool and refreshing; her window opened upon a little strip of garden, whose shrubs and vegetables looked almost home-like after the plains. It was strange what a difference even this slight elevation—only fourteen hundred feet above Delhi—made in the temperature. Sheer fatigue gave her sleep that night, and at ten o'clock next morning she was once more on her way in the tonga, a strongly-built two-wheeled vehicle, like a low dogcart with a roof upheld by iron bars. Kate took her place in front beside the wild-looking charioteer, with her ayah on the back seat among the small quantity of baggage which the conveyance could hold besides the passengers. Her other attendant she left to bring on the remainder of her property by bullock train.

In spite of herself she enjoyed the drive. They travelled fast, for the ponies were changed every four or five miles, and the driver was anxious to earn the memsahib's backsheesh. The narrow path lay between lofty hills for a space, then wound like a snake along the steep mountain's face, anon became a shelf cut in the living rock, whose edge, a loose wall of stones protected from the fathomless abyss below. Far away down in the ravines Kate could see men toiling in their terraced gardens and tending their herds of diminutive cattle. So distant were they that the kine might have been dogs. The road had its sights, too. Now a long string of laden camels tied nose to tail came lurching along with ungainly strides, blocking up the road, and forcing the tonga-driver to pull up until the camel men urged the stupid beasts aside with blows and cries. Now the discordant notes of a bugle echoed and reverberated among the hills, and the mail gharry from Simla came thundering round the bend at a gallop, while the driver blew with all his might a warning to travellers to draw aside and let him pass.

The day wore on, and after a stoppage at the dâk bungalow for tiffin, they pushed on again. The fresh, invigorating perfume of the pine-like deodars and the rapidly lowering temperature told Kate that she had passed into cool altitudes at last. Before long the lonely cantonment of Dugshai, perched on the summit of a rocky crag, came in sight, and was lost to view again as the tonga swept along the serpentine road. A fleeting glimpse of the distant snows formed a fitting background for the rugged grandeur of the Himalayan landscape, and the solitary majesty of the mountain crests held Kate spellbound, as though by gazing she could bring their secrets nearer.

She broke the long drive of fifty-eight miles at Solon, where she slept the night. And on the following afternoon, the road suddenly emerged from behind a towering wall of rock, and showed her the great hill capital stretching like a crescent across Jakko's concave breast. It was still far away; hill and valley lay between them, and she could only discern the houses as white dots against the dark green woods; but two hours later she had reached the depot, and was following a guide to The Rockcliff Hotel.

It was a large straggly building, or rather collection of buildings, hanging to, rather than standing on, the steep slope below the main road of the station. Kate looked out of her front balcony clear over the tops of the oaks and deodars, while the back door of the bathroom opened upon the rock itself.

She stood for a few minutes drinking in the cold evening breeze, drawing her cloak more closely round her. From the balcony she had an unbroken view over the lower-lying station of Subathu to the plains fifty miles away, which looked like a faintly drawn map, whereon light and shade marked towns and fields. A well-known voice made her start and glance downwards.

"I knew it was Katie; I was just sayin' to Colonel Fairbrother that it was you, Katie. How are you, my dear? I'll come up to your room at once." The speaker was Mrs. Macdonald, whose fat, good-tempered face was aglow with pleasure. "And you've left Bairdsley down at Delhi," she said as she released Kate from an embrace resembling the hug of an affectionate bear. You're looking very ill, my dear, and it's no wonder, after all your trouble. Tea's ready in my room, so you will just come straight in and have some."

Kate followed her friend, and was soon ensconced in a deep armchair before a blazing wood fire, which roared and crackled on the grateless stone fireplace.

"Are there any more old Meerut friends here besides yourself, Mrs. Macdonald?" she asked, as that kindly woman took a seat near her.

"Not one, my dear—not one. The Strachans are away to England on two years' furlough; and the Allertons have gone to Mussoorie again. And, Katie, believe me, Mussoorie is a better place than Simla. There's too much kow-towing to the burra sahibs here for my taste; the very shopkeepers smack of officialdom; but Bairdsley will have told you all about it. You'll not be going about to the gaieties here just now, but you'll need to hire a 'rickshaw and a crew of jampannies. Rascals they are, my dear, as you'll soon know. Mine asked an extra rupee a month because they said I was so heavy to drag up the hills. Impudence!" And Mrs. Macdonald laughed pleasantly at the recollection of her candid jampannies' conduct; she was incapable of taking offence, even at such an illusion to her increasing size. She rattled on in a lively strain for half an hour, and then stopped short to tell Kate that she was looking dead-tired, and must go to bed as soon as she had had her dinner.

"You made two dâks of it from Kalka, I suppose? but it's a tiresome journey anyway in those rattling tongas."

Kate was well pleased to escape to her own apartments, and eat her dinner alone. The hotel was full, and the khitmugars belonging to the establishment were few in number, so it was a long, wearisome matter to get dishes removed and brought up. When she had done, she sat down to write to her father, but soon found that she was unable to keep her eyes open, and abandoned the business until the following day; although the postponement would entail a week's delay.

The next morning was spent in hiring a 'rickshaw—a species of two-wheeled perambulator, drawn and pushed by four men, styled "jampannies;" and in the mysterious rites which constitute "settling down" in new quarters. Mrs. Macdonald came up to see her after breakfast, and gave her a large fund of information about Simla, its population and their ways.

"You see, Katie, no one but Mrs. Viceroy and Mrs. Commander-in-Chief are allowed to drive carriages up here. Lady Turnbull, that's Mrs. Lieutenant-Governor of the Punjaub, has one too, but those three are all there are. You can get saddle-horses on hire for forty rupees a month, with syce and feeding in; but you'll pay an extra rupee a month for water. I'm going to a picnic to-day given by the Fardells, out at Mashobra, and I'd take you if you'd come. It's a great place for picnics this; the Bardhams gave one last Monday on the top of Jakko, and how I ever got up there I don't know. You must wear a solah topee if you're going out during the day. It's cold up here, but the sun's as strong as ever it is in the plains; young Mr. Scott had a sunstroke only yesterday, by riding out to make calls in a small hat.

Kate was relieved when the hour compelled her loquacious friend to go and prepare for the picnic; and, as soon as she was alone, set to work to write to her father. She could not keep what she had learned from that letter in her own breast, and even though her despatch must lie for a week in the post-office she would feel more at ease when she had committed the story to paper and asked for advice. She had never heard of the Miss Carsworth whose fortune she was to inherit. Dr. Harrison must at least know of her, though, and he would be able to tell her if what Mr. Robert Anderson had written to her husband was true. George's uneasiness at her father's proposal to go to the Riviera was explained by that letter. She had not referred to it in writing, but earnestly hoped that the Doctor would take steps to become acquainted with his relative, if he did not know her already.

Time passed very slowly for her in Simla. Even had she not been able to plead mourning for her abstention from parties she would not have gone to them. She joined the library, and spent the days reading or in the society of one or two ladies who lived at The Rockcliff and did not give themselves up to social amusements quite so exclusively as the rest. She soon grew tired of the 'rickshaw, and as soon as her baggage and saddlery arrived, hired a steady hill pony, upon which she made long solitary excursions as far as the roads allowed her to go. The rides about Simla were not numerous; round Jakko; out to Mashobra, or Jutogh; down through the woods which hemmed in Annandale, the Simla racecourse, and one or two by-paths, steep as the roof of a house, completed the list. Her health gradually improved, and her pallor fled after a few weeks upon the hill; and Mrs. Macdonald told her she was more like the Kate who came out fresh from home, now, than she had ever been since.

"Do you hear from Bairdsley often, Katie?" she enquired one day, about a month after the young lady's arrival.

"Oh, yes, I hear from him," replied Kate, with a faint blush. She had had one brief note from him enclosing a cheque since she left Delhi. He readdressed and sent on her father's letters, but beyond that they held no communication whatever.

"Of course, he has told you that the 110th is ordered to Peshawur?"

Kate's look of surprise answered Mrs. Macdonald, and she continued: "Colonel Collins, the assistant quarter-master general, told me yesterday that the regiment was going at once. I thought Bairdsley would have telegraphed you."

"He hasn't done so yet, but I've no doubt he will," she replied, wondering as she spoke whether her husband would feel himself called upon to inform her of his movements.

"Just wait a day or two. No doubt his letter is on the way, since he hasn't wired. He will be up to his eyes in work, poor man."

But two days fled, and three, and four, and never a line came from Bairdsley to his wife. Kate wrote to him at last to enquire what he was doing, when he expected to leave Delhi, and whether there was any prospect of returning. Now that they were parted by distance, she could remember and fulfil such of a wife's duties as she might, without that feeling of revulsion which had forced her to escape from his society. She must know what his movements were, in order to hide their feud; she was his wife, after all, and it was not desirable that all the world should know how they stood towards one another. So she wrote, and received no answer for three weeks. Then she received one from him at Peshawur. It told her that he should not be able to write to her again, for a considerable time, as he was attached to a column whose mission was to proceed into the hill country across the frontier, and exact submission from or inflict punishment on a warlike hill tribe, who had been raiding in British territory. The column would be absent for a month or six weeks; but he would write when he returned.

Bairdsley had decided that nothing was to be gained by quarrelling, and that his policy now must be one of conciliation. He therefore wrote his letter in a tone of respectful contrition which had an air of sincerity that was not without its effect upon his wife. Kate had been far too deeply wronged to forgive, but she was not vindictive. Her conscience pricked her that she felt too little wifely anxiety for his safety in this dangerous guerilla warfare in which he was about to engage.

(*To be continued.*)

